# Reversible Inferolateral ST-Segment Elevation Associated with Small Bowel Obstruction

**DOI:** 10.1155/2017/5982910

**Published:** 2017-03-30

**Authors:** Ankit Upadhyay, Sudheer Chauhan, Umair Jangda, Vipul Bodar, Ahmed Al-Chalabi

**Affiliations:** Department of Medicine, Jamaica Hospital Medical Center, Jamaica, NY 11418, USA

## Abstract

ST-segment elevation is an important and alarming electrocardiographic sign that necessitates immediate attention but does not always indicate that the primary pathology is cardiac in origin. It needs to be interpreted in the clinical context as several pathological conditions involving especially gastrointestinal tract may lead to delayed diagnosis and treatment as well as complications from invasive unnecessary interventions. We present two patients, a 64-year-old male and a 71-year-old female, who were admitted to the emergency room of a community-based hospital with similar complaints of worsening epigastric abdominal pain and were diagnosed later with small bowel obstruction. Both patients reported a history of abdominal surgeries in the past. Also in both patients the ECG showed signs of ST-segment elevation in inferior and lateral leads. These ECG changes were related to the intra-abdominal pathology as no evidence of contributing coronary artery disease could be found. In addition, prompt resolution of ST-segment elevation was seen after surgical treatment. The pathophysiological etiology of electrocardiographic changes accompanying small bowel obstruction is yet to be explored.

## 1. Introduction

Electrocardiography (ECG) is widely used tool in the screening, diagnosis, and management of cardiac diseases in the emergency room setting because it is easily available, simple, noninvasive, and less expensive. ST elevation on electrocardiography has various clinical implications with the most common being acute myocardial infarction, a medical emergency that requires immediate intervention to salvage myocardial tissue and reduce mortality. Pathological conditions involving the gastrointestinal system can present with similar ECG abnormalities, which may lead to misdiagnosis and complications from delayed or unnecessary treatment. We present two cases of patients developing electrocardiographic changes of ST elevation caused by intestinal obstruction.

## 2. Case Presentations

### 2.1. Case  1

**Figure 1:**
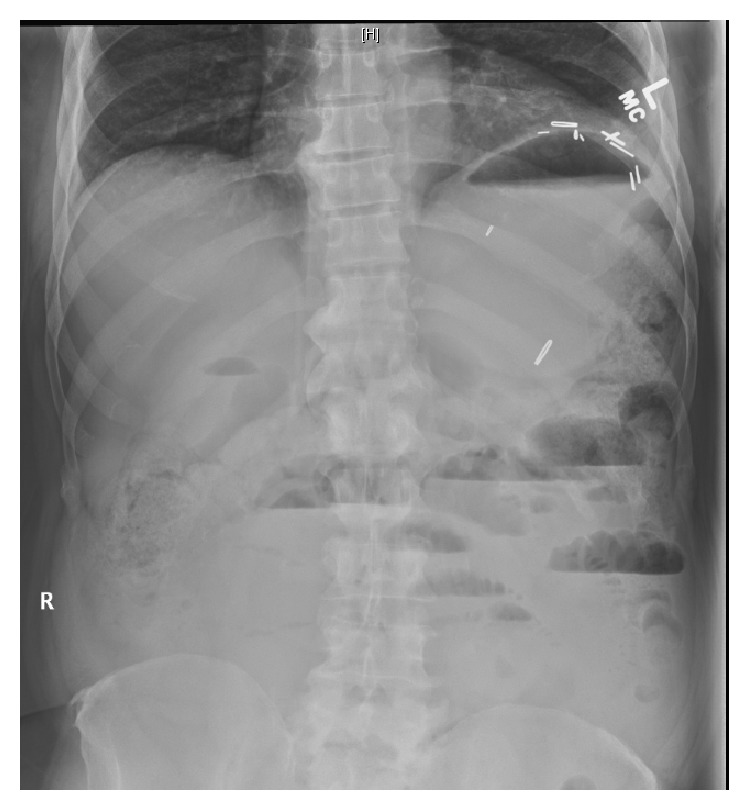
Abdominal X-ray showing scattered air-fluid levels in minimally prominent small bowel loops (case  1).

**Figure 2:**
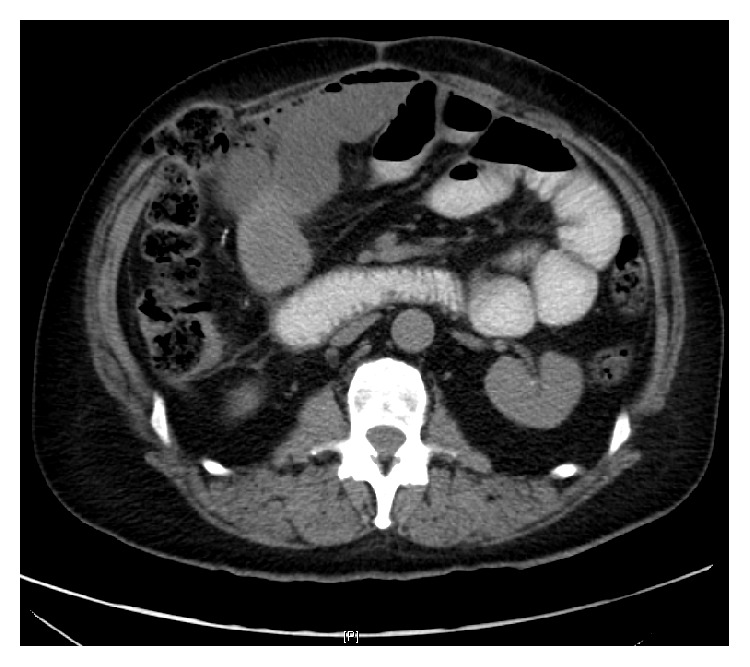
Dilated multiple loops of proximal small bowel with collapsed distal loops of small bowel consistent with small bowel obstruction (case  1).

**Figure 3:**
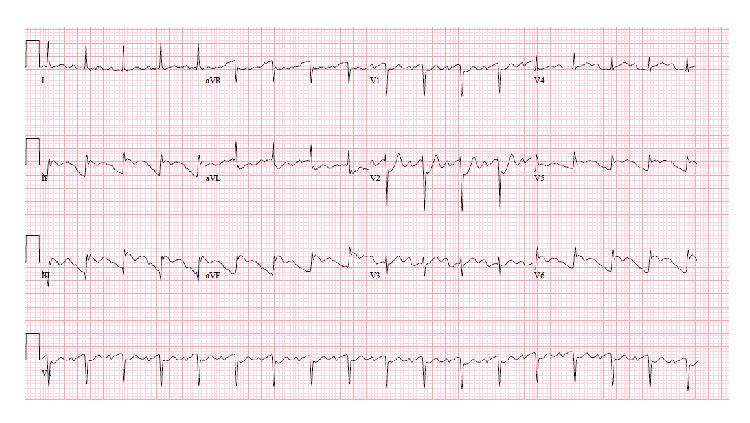
ST elevation in inferior and lateral leads (case  1).

**Figure 4:**
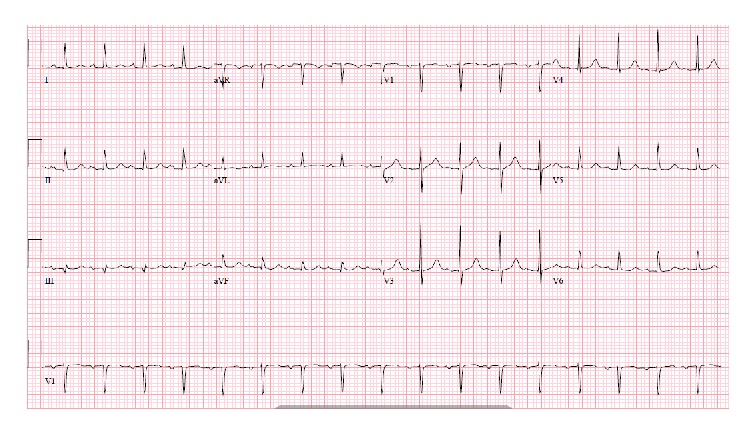
Resolution of ST elevation in inferolateral leads after surgical decompression (case  1).

A 64-year-old male presented with worsening abdominal pain, constipation, nausea, and vomiting for the last 3 days. He denied chest pain, palpitations, dizziness, shortness of breath, or loss of consciousness. He has been passing hard stool and small amount of gas. His past medical history was remarkable for diabetes mellitus, hypertension, and chronic kidney disease. He had previous multiple abdominal surgeries following chronic alcoholic pancreatitis, which included partial pancreatectomy, left hemidiaphragm repair, and colostomy with subsequent reversal. On physical examination, the patient was lethargic and dehydrated. He had multiple well healed surgical scars on a distended and tender abdomen. Bowel sounds were high pitched. Scattered air-fluid levels indicating small bowel obstruction were seen on erect abdominal X-ray (Figure 1). Computer tomography confirmed the diagnosis (Figure 2). To our surprise, the ECG showed sinus tachycardia with inferolateral ST elevation (Figure 3). However, troponin was 0.048 ng/mL (just above upper normal limit) and CKMB was normal. Serum Creatinine was 2.4 mg/dL. Liver enzymes and lactate were within normal limits. Emergent echocardiography was done and it revealed an ejection fraction of 75% with no regional wall motion abnormalities. Patient was then referred for surgical treatment for intestinal obstruction. Soon after laparotomy and resolution of obstruction, the dramatic ECG changes disappeared (Figure 4) while the cardiac biomarkers remained negative making an acute coronary event unlikely.

### 2.2. Case  2

**Figure 5:**
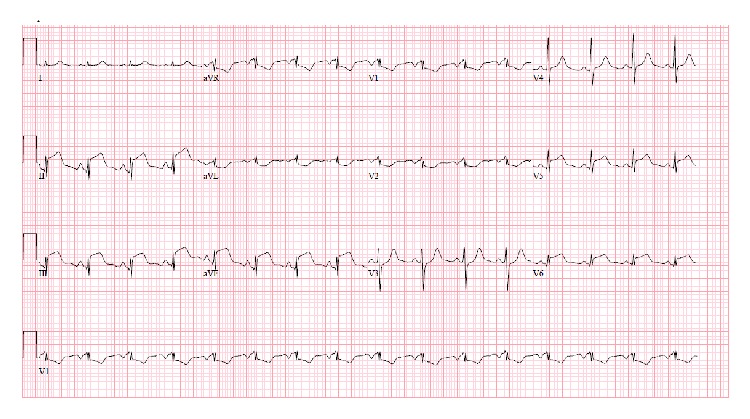
ST elevations in leads II, III, aVF, V5, and V6 (case  2).

**Figure 6:**
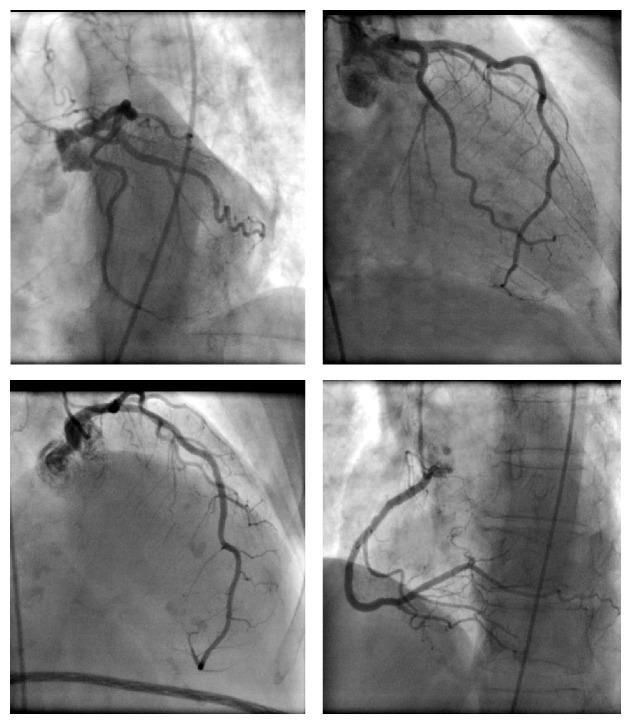
Coronary angiography showing no obstructive coronary artery lesions responsible for ST elevation (case  2).

**Figure 7:**
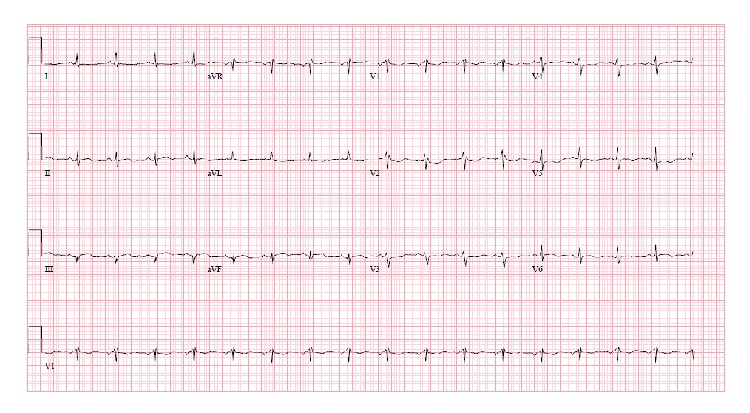
Resolution of ST elevation after surgical intervention (case  2).

A 71-year-old female patient presented with epigastric pain in the last 12 hours. The pain was moderately severe, intermittent, and not associated with nausea and vomiting. She did not have any bowel movement for last two days but was passing some flatulence. She denied fever, chills, recent viral infection, chest pain, palpitation, shortness of breath, and dizziness. She had no previous history of heart disease, diabetes mellitus, and hypertension. She never smoked before. In addition to cholecystectomy and a cesarean section, her past surgical history was significant for a right sided femoral hernia that remained asymptomatic for the last 10 years. On physical exam, the abdomen was soft, nontender, nondistended, and without hepatosplenomegaly and right femoral hernia was partially reducible. Laboratory results revealed leukocytosis with WBC of 14,000, normal electrolytes, hepatic enzymes, and renal function. However, in the emergency room, ECG demonstrated ST elevations in leads II, III, aVF, V5, and V6 (Figure 5). Patient was transferred to cardiac catheterization lab for emergent angiography via left femoral approach because of right sided hernia. This revealed no angiographic evidence for obstructive coronary artery disease (Figure 6) but an abnormal left ventricular systolic function with ejection fraction around 35%. Diaphragmatic and posterobasal hypokinesis was evident on contrast ventriculography. Serum troponin levels were elevated up to 5 ng/mL which trended down to normal level later on. After myocardial infarction has been ruled out, patient was evaluated by surgery team and hernia was found to be irreducible at that time. Subsequently, patient underwent exploratory laparotomy, which demonstrated strangulated, nonperforated ischemic segment of small intestine in right femoral hernia with dilated proximal small intestine. Eight centimeters of small bowel was resected followed by establishing a side-to-side (functional end-to-end) anastomosis. Repeat ECG immediately after the surgical procedure revealed resolution of ST elevation in inferior and lateral leads (Figure 7). Few days later, patient was discharged on Lisinopril and Carvedilol. After six months of follow-up, echocardiogram revealed normal left ventricular size and systolic function without regional wall motion abnormalities.

## 3. Discussion

A number of cardiac and noncardiac conditions that mimic ST elevation myocardial infarction have been described in the current literature. Coppola et al. described myriad of disorders causing ST elevation including cardiac causes such as pericarditis, myocarditis, Brugada's syndrome, aortic dissection, Prinzmetal's angina, Takotsubo (stress-induced) cardiomyopathy, and hypertrophic cardiomyopathy; pulmonary causes such as pulmonary embolism, pneumothorax, and atelectasis; gastrointestinal causes like cholecystitis, pancreatitis; and other conditions like drug induced, hyperkalemia and hemorrhagic cerebrovascular disease [1]. To our knowledge, there are only three cases describing the ST-segment elevation in acute intestinal obstruction and very few cases reporting ST elevation due to other gastrointestinal pathology such as esophageal perforation and gastric tube insertion and inflation [2, 3]. In the first case, the patient had history of abdominal surgeries and presented with obvious symptoms of intestinal obstruction so we interpreted the ECG changes as a reflection of the intra-abdominal pathology and avoided invasive coronary angiography. Also pulmonary embolism was low in the differential diagnosis but there were no supportive evidence of right ventricular decompensation on echocardiography. In the second case, diagnosis was challenging and, hence, the patient underwent coronary angiography to rule out acute coronary syndrome. Although the possible presence of atypical Takotsubo cardiomyopathy contributing to the clinical presentation may explain the ECG and angiography findings, especially ST elevation pattern deriving from a prominent S in II, III, and aVL, the patient had a clear evidence of intestinal obstruction. Therefore, we believe that ECG changes were also related to the acute intestinal pathology. Though it was not performed, cardiac MRI would have added valuable diagnostic information. The absence of gadolinium late enhancement would have supported the hypothesis of an extracardiac mechanism in case  1 and in case  2 it would have helped to exclude myocarditis.

The underlying pathophysiology of ST elevation in intestinal obstruction is still unknown. It has been postulated that distension of hollow organs like the stomach, the gallbladder, or even reconstructed gastric or jejunal tubes following esophageal resection might cause direct cardiac compression leading to electrophysiological changes presented as ST-segment abnormalities [2, 3]. In our cases, increased intra-abdominal pressure might have led to relative displacement or compression of the inferior surface of the heart resulting into change in the mean QRS axis and/or voltage [4–6]. An alternative hypothesis is that the distension of gastrointestinal organs might lead to an enhanced vagal tone with vasovagal reflex and resultant disturbance of ventricular depolarization. Interestingly, the full stomach test has been proposed as a diagnostic tool for Brugada syndrome as the characteristic ST-segment elevation can be potentiated by a full meal [7]. Finally, a mechanism similar to that underlying Takotsubo cardiomyopathy with resultant catecholamine-induced microvascular spasm or dysfunction with myocardial stunning may explain the transient ST-segment elevation and recovery of left ventricular function in estrogen deficient women in our second case [8]. Therefore, we think that pathophysiologic process of ST elevation in intestinal obstruction does not simply follow one pathway or can be multifactorial.

## 4. Conclusion

ECG still remains the first study of choice for screening patients for cardiac diseases in the emergency room. Broad differential diagnosis should be kept in mind for ST elevation seen in ECG particularly in absence of ischemic symptoms. If misinterpreted, these ECG changes may lead to unnecessary and invasive interventions while delaying appropriate treatment. These two cases further broaden the list of gastrointestinal disorders causing ST elevation on ECG.
